# Brachial Artery Coiling: Report of a Rare Case

**DOI:** 10.7759/cureus.2603

**Published:** 2018-05-10

**Authors:** Iva N Dimitrova, Georgi P Georgiev

**Affiliations:** 1 Department of Cardiology, University Hospital St. Ekaterina, Medical University of Sofia, Bulgaria, Stara Zagora, BGR; 2 Department of Orthopaedics and Traumatology, Medical University of Sofia, Bulgaria, University Hospital Queen Giovanna, Sofia, BGR

**Keywords:** brachial artery coiling, heart catheterization failure

## Abstract

Nowadays, the transradial approach is increasingly used for performing percutaneous coronary interventions and is preferred over the transfemoral approach. In the latest European Society of Cardiology guidelines (2017) for management of acute myocardial infarction in patients presenting with ST-segment elevation, the transradial approach is recommended over the transfemoral one if performed by an experienced radial operator (MATRIX study). Transradial procedure failures may be related to puncture failure, artery spasm, or to anatomical variations that require specific catheter handling or changing with a contralateral or transfemoral approach. Herein we report a failure of transradial heart catheterization due to brachial artery coiling.

## Introduction

The brachial artery (BA) is a continuation of the axillary artery and starts at the lower margin of the teres major muscle and ends about 1 cm beyond the elbow joint where it divides into the radial and ulnar arteries [1]. Tortuosity along its course could impede procedures like transradial (TR) catheterization or thrombectomy of the BA occlusion [1-3]. Moreover, the BA could make a full circle of 360 °, termed as “coiling”. The estimated frequency of this rare condition is below 1 % [4].

Herein we describe a case of BA coiling found during a TR catheterization in an 81-years-old-female with clinical and electrocardiography signs of non-ST-elevation myocardial infarction (non-STEMI). Due to this anatomical variation, a failure of TR heart catheterization was reported.

## Case presentation

**Figure 1:**
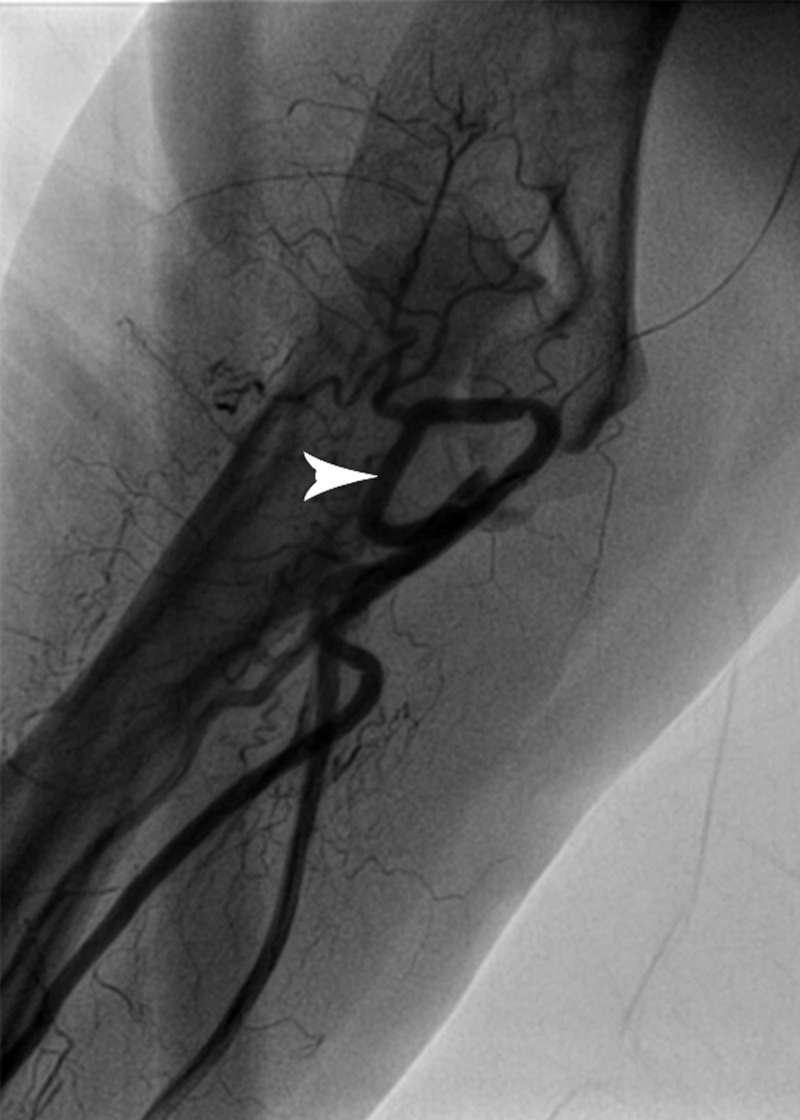
Arteriography of the right arm presenting brachial artery coiling (arrowhead)

An 81-years-old female presented in the emergency room of our hospital with clinical and electrocardiography signs of non-STEMI. A coronary angiogram was planned. Allen's test was performed and the TR approach was chosen. The radial artery was successfully accessed with a 6F radial sheath. A 5F Tiger catheter (Terumo Corporation, Somerset, New Jersey) was introduced through a 0.035 guide wire, but we felt resistance in the wire progression at the level of the cubital region. The wire was removed and a retrograde contrast injection was given to visualize the obstruction. We found a loop of 360 degrees of the BA just proximal of the bifurcation with initial spasm (Figure 1).

A 0,035 hydrophilic coated guidewire passed through, but the catheter advancement was difficult and accompanied with pain. Thereafter, we used an alternative transfemoral (TF) access with a successful outcome. A critical left anterior descending artery stenosis was successfully treated with a drug-eluting stent implantation.

## Discussion

Coiling is frequently described in  the internal carotid artery as a cause of cerebrovascular insufficiency or is connected with carotid atherosclerosis. Coiling of the other arteries is rarely reported [2]. The first clinical report of BA coiling have been described by Casten and Forman in 1962 [5]. According to Ilijevski, et al. [2] this anomaly is seldom reported because it commonly remains asymptomatic. The variant artery is reported in cases of iatrogenic injury, occlusion, or is found accidentally in cases of angiography for monitoring purposes. Coiling of 360 degrees could simulate a painless pulsating mass at the brachial region and could be mistaken for an aneurysm of the BA [4]. In such cases, a duplex Doppler sonography or multidetector computed tomography could reveal the proper diagnosis [1,4].

In rare cases, different anatomical variations, such as BA coiling, could lead to differences and failures in the TR approach [6]. In our case, due to excessive BA coiling and arterial spasm, the catheter advancement was difficult and was accompanied with pain. Therefore, we preferred to use an alternative TF approach to perform a drug-eluting stent implantation for critical left anterior descending artery stenosis. Trying to pass in such a BA coilng is painful and associated with increased spasm and risk of artery perforation.

The etiology of BA coiling is unknown. According to Hsu, et al. [4] and Paulsen, et al. [7], it is similar to that of the carotid artery and is associated with aging and is the result of hypertrophy and elongation of the wall of the artery. Paulsen et al. [7] proposed that coiling is exacerbated by arteriosclerosis or fibromuscular dysplasia. Coiling of the artery may produce luminal narrowing, which can lead to turbulent blood flow and subsequent intimal ulceration and embolization [8].

According to Jelev and Guirov [1], two types of BA could be involved in coil formation. In the first type, the common BA in its distal part is implicated, and in the second type, coiling could affect the superficial BA.

## Conclusions

In conclusion, interventional cardiologists should expect and be familiar with different arterial anatomical variations found during the TR approach. Knowledge of these variations will increase their learning curve, and thus help in avoiding potential complications. In the reported case, due to the high risk of BA perforation, an alternative TF approach was used.
